# Early erratic flight response of the lucerne moth to the quiet echolocation calls of distant bats

**DOI:** 10.1371/journal.pone.0202679

**Published:** 2018-08-20

**Authors:** Ryo Nakano, Andrew C. Mason

**Affiliations:** 1 Department of Biological Sciences, University of Toronto Scarborough, Toronto, Ontario, Canada; 2 Division of Fruit Production and Postharvest Science, Institute of Fruit Tree and Tea Science, National Agriculture and Food Research Organization, Tsukuba, Ibaraki, Japan; AgroParisTech, FRANCE

## Abstract

Nocturnal insects have evolved ultrasound-sensitive hearing in response to predation pressures from echolocating insectivorous bats. Flying tympanate moths take various evasive actions when they detect bat cries, including turning away, performing a steering/zigzagging flight and ceasing flight. In general, infrequent ultrasonic pulses with low sound intensities that are emitted by distant bats evoke slight turns, whereas frequent and loud ultrasonic pulses of nearby bats evoke erratic or rapid unpredictable changes in the flight path of a moth. Flight cessation, which is a freezing response that causes the moth to passively dive (drop) to the ground, is considered the ultimate last-ditch evasive behaviour against approaching bats where there is a high predation threat. Here, we found that the crambid moth *Nomophila nearctica* never performed passive dives in response to frequent and loud ultrasonic pulses of >60 dB sound pressure level (SPL) that simulated the attacking echolocation call sequence of the predominant sympatric insectivorous bat *Eptesicus fuscus*, but rather turned away or flew erratically, regardless of the temporal structure of the stimulus. Consequently, *N*. *nearctica* is likely to survive predation by bats by taking early evasive action even when it detects the echolocation calls of sympatric bats hunting other insects at a distance. Since aerially hawking bats can track and catch erratically flying moths after targeting their prey, this early escape strategy may be common among night-flying tympanate insects.

## Introduction

Nocturnal insects are exposed to intense predation pressure from insectivorous echolocating bats [1,2], which has led to the evolution of ultrasound-sensitive ears and escape behaviours [3–5]. Many moths possess tympanal hearing organs and respond to ultrasonic pulses with diverse evasive actions [6]. In general, ultrasound with a relatively low intensity and infrequent pulse repetition rate causes flying tympanate moths to turn away from the sound source [7,8], whereas loud and frequent ultrasonic pulses evoke erratic responses, including looping, steering and zigzagging flight or even dropping and diving to the ground. Dropping and diving are the ultimate last-ditch evasive behaviours that are performed when moths detect the approach- and terminal-phase pulses of approaching bats [9,10], and are often exhibited by noctuid moths (Noctuoidea) and geometrid moths (Geometroidea) [7–11]. In addition, tiger and lichen moths (Erebidae; formerly Arctiidae), the beggar moth *Eubaphe unicolor* (Geometridae) and male hawk moths (Sphingidae) are able to actively generate sound, which can function as a bat-avoidance tactic by warning bats and jamming their echolocation signals [12–18].

It is widely believed that bats in pursuit fail to catch moths that initiate erratic flight shortly before contact due to their unpredictable flight path [1]. Recently, however, it was reported that moths that attempt to escape a bat in direct pursuit are caught approximately 37% of the time, with the success depending on the moth’s radial acceleration and the angle of its evasive flight [10]. Considering the multiple attacks a bat makes in a night and the number of flights an adult moth undertakes during its life, it stands to reason that prey insects that are attempting to dodge predatory bats in pursuit face a high risk of capture [18,19]. Exceptions to this are insects flying close to the ground or into vegetation, because some bats abort their pursuit of target prey under these conditions to avoid collision [19–22]. For example, although the big brown bat, *Eptesicus fuscus* (Palisot de Beauvois) (Chiroptera: Vespertilinoidae), which is the focus of the present study, sometimes flies into vegetation to capture buzzing beetles [23,24], more than 50% of trained bats abort and/or do not attempt to pursue silent prey that are flying close (within 10 cm) to a cluttered background, i.e. plants [22]. Consequently, if moths begin their evasive actions and hide in vegetation before bats start to chase them, the bats would not be able to lock on to them, reducing the predation threat within the bats’ reaction range. Since predacious bats use highly directional, loud (>120 dB sound pressure level (SPL) at 10 cm) ultrasonic pulses to hunt prey in the air [25–27], the early detection and avoidance of distant echolocating bats are the best strategies for nocturnal tympanate insects [1,2,14]. However, although much is known about the early turning-away response to distant bat echolocation calls in Noctuoidea and Geometroidea [7–9,11], little research has been conducted on other tympanate moths.

Here, we investigated early evasive manoeuvres in the lucerne moth, *Nomophila nearctica* Munroe (Lepidoptera: Crambidae), which possesses a pair of abdominal tympanal ears that are sensitive to 30–100 kHz [3,28] and is mainly distributed in North and Middle America, where it is a known minor pest in grasslands of clover, alfalfa, celery, soybean and corn [29]. In Ontario, Canada, *N*. *nearctica* is currently sympatric with *E*. *fuscus* and historically also occurred alongside the little brown bat, *Myotis lucifugus* (LeConte) (Chiroptera: Vespertilinoidae) [28,30]. These moths can fly at high for long-distance migration from southern to central/northern parts of America in autumn, during which time they are consumed by the Brazilian free-tailed bat, *Tadarida brasiliensis* I. Geoffroy (Chiroptera: Molossidae), which is a high-altitude insectivorous species in Texas [31]. During the daytime in summer, however, we readily found the moths on low vegetation, implying that they fly close to the ground when not migrating. Therefore, since *E*. *fuscus* is known to prey on insects flying near to the ground [23], non-migrating *N*. *nearctica* would be exposed to predation pressure from this bat. To confirm the overlap in diel activities of *N*. *nearctica* and *E*. *fuscus*, we estimated the foraging time of *E*. *fuscus* and the active times of *N*. *nearctica*. We then examined the incidence and mode of evasive behaviours exhibited by tethered flying moths in response to simulations of the temporal structures of echolocation pulses recorded from wild *E*. *fuscus*.

## Materials and methods

### Sampling and rearing of moths

We caught wild adult *N*. *nearctica* with an insect net in the daytime at the campus of the University of Toronto Scarborough (43°78′10″N, 79°18′36″W; Toronto, Ontario, Canada) from June to August 2014 and 2015. Larvae derived from gravid females were reared on an artificial diet (Silk-mate™ 2M; Nosan Corp., Yokohama, Japan) and kept at a constant temperature of 21 ± 1°C under a 16:8 h light:dark (L:D) cycle until they were used in the experiment. Male and female moths were sexed on the day of emergence (0 days old), and housed in 30 × 30 × 30 cm nylon mesh cages with a water supply. Ethical approval was not required for this study.

### Recording and analysis of bat echolocation calls

In early August 2014, we manually recorded the echolocation calls of *E*. *fuscus*, which is a dominant bat species at the site where we captured *N*. *nearctica* [28]. Recordings were made using an UltraSoundGate 416–200 (Avisoft Bioacoustics, Berlin, Germany) with a CM16/CMPA condenser microphone and Avisoft-RECORDER USGH 4.2.18 software (250-kHz sampling rate; 16-bit; .wav file format). Before making the manual recordings, we monitored bat activity using a portable bat detector (D240X; Pettersson Elektronik, Uppsala, Sweden) in heterodyne mode. We then measured the temporal characteristics of the pulse duration and inter-pulse interval (duration of silence between pulses) in the search, approach and terminal phases of the echolocation call sequences, according to the previously defined predation sequence [32]. The start and end points of a pulse were determined by discriminating sounds of >1 dB from the background noise with an oscillogram and spectrogram using BatSound 4.03 software (Pettersson Elektronik).

### Active times of bats and moths

To estimate the foraging times of echolocating *E*. *fuscus*, we used a D500X ultrasound detector (Pettersson Elektronik) to automatically record wild bat echolocation calls from 1 h before sunset (around 20:00) to 1 h after sunrise (7:00) from early June to late July 2014. These recordings were made at a sampling rate of 300 kHz (16-bit; .wav file format) for 5 s, including a 1-s pre-trigger buffer. In June–July 2015, we used a digital voice recorder with a voice-activated recording function (WS-822; Olympus Corp., Tokyo, Japan) that was connected via a 3.5-mm earphone jack to a heterodyne bat detector set at 30 kHz (Bat4 Bat Detector; Magenta Electronics Ltd., Tutbury, UK) to obtain 5-s-long recordings. We then analysed the time stamps of the .wav call files.

To quantify the locomotion of *N*. *nearctica* in a given day, we introduced 2- to 3-day-old virgin moths (16 males and 18 females) individually into a transparent plastic vial (90 mm high × 44 mm internal diameter), the inside walls of which had been partially sprayed with water, under the same temperature and photocycle conditions as were used during rearing. We then recorded their movement using a locomotion monitor with nine infrared beams per tube (LAM 60; Trikinetics Inc., Waltham, MA, USA) connected to DAMSystem308 software (Trikinetics Inc.) on a laptop PC. To calculate moth nocturnality (%), we first converted the individual count data (the number of passes) obtained per 10 min to binary data (0 or 1), so that any locomotion detected in a 10-min period was treated as ‘1’ regardless of the number of passes. Since a 16L:8D photocycle was used in the experimental room, we doubled the converted values for the scotophase (8-h dark phase) and then calculated the proportion of locomotion detected during this period.

### Ultrasound stimulus

To determine which pulse structures and sound levels of hunting bat echolocation calls cause evasive manoeuvres in *N*. *nearctica*, we synthesized six types of pulses with 25% rise/fall times based on echolocation calls recorded in the field using Audacity® 2.1.0 software (Audacity Team, USA; 192-kHz sampling rate; 16-bit; .wav file format). These pulses were composed of 30-kHz pure tones for simplicity. The temporal structures of the six pulse types were as follows: 1) search phase pulse with a 10-ms pulse duration (PD) and 111-ms inter-pulse interval (IPI) (8.3 pulse s^−1^); 2) early-approach phase pulse with an 8-ms PD and 88-ms IPI (10.4 pulse s^−1^); 3) middle-approach phase pulse with a 6-ms PD and 66-ms IPI (13.9 pulse s^−1^); 4) late-approach phase pulse with a 4-ms PD and 40-ms IPI (22.7 pulse s^−1^); 5) early-terminal buzz with a 2-ms PD and 13-ms IPI (66.7 pulse s^−1^); and 6) middle-terminal buzz with a 1-ms PD and 5-ms IPI (166.7 pulse s^−1^). The late-terminal buzz, which has a <0.5-ms PD and 5-ms IPI (>181.8 pulse s^−1^), was not simulated in this study due to the limited sampling rate not allowing accurate simulation of the transient pulse as well as the lack of opportunity for a flying moth to escape an extremely close chasing bat [9,10,12,32–38].

Each ultrasound stimulus was broadcast for 2 s at 40, 50, 60, 70, 80, 90 and 100 dB SPL (re. 20 μPa) from an electrostatic speaker (ES1 speaker connected to an ED1 speaker driver; Tucker-Davis Technologies, Alachua, FL, USA) positioned 20 cm away from a tethered, resting moth (see below). The stimuli sound levels were confirmed with a 1/4-inch microphone (type 4939; grid-off, connected to a type 2670 preamplifier and a type 2690 Nexus™ conditioning amplifier with 0.02–140-kHz band-pass filter; Brüel and Kjær, Nærum, Denmark) by referring to a known signal voltage from a sound calibrator (type 4231; 94 dB SPL at 1 kHz; Brüel and Kjær).

### Moth avoidance behaviour

We examined the frequency and mode of avoidance manoeuvres exhibited by *N*. *nearctica* in response to simulated echolocation pulses of *E*. *fuscus* using 2- to 3-day-old virgin moths of both sexes (49 males and 47 females). Individual moths had a fine nylon fishing line tied around their cervix and were suspended from a metal rod attached to the side wall of the experimental room, which stabilized their flight in a restricted space. These tethered moths generally started to fly when a small piece of paper that served as a platform was removed from beneath their legs, with those moths that did not exhibit flight being removed from the experiments. Immediately after stable flight had been confirmed, the ultrasound stimuli were presented to each tethered flying moth in a random order, with different stimuli being broadcast to the same moth at ca. 20-min intervals to avoid sensory behavioural fatigue. Moth behaviour was then recorded with a digital high-definition (HD) camcorder in infrared recording mode (HDR-PJ760V; Sony, Tokyo, Japan) connected to a heterodyne bat detector (Magenta Electronics Ltd.) that was set at 30 kHz to monitor the ultrasound stimuli presented. Recordings were made for at least 3 s from >1 s before the broadcast of the 2-s ultrasound stimulus to the end of the broadcast. All experiments were carried out in the scotophase under a dim red light and behavioural responses observed during the stimulus presentation were analysed blindly.

The incidence of avoidance responses was analysed with likelihood-ratio (LR) tests in cumulative link mixed models (CLMM) using the ‘ordinal’ package in R 3.2.4. In these models, three conventional flying insect escape response behaviours to bat echolocation [1,2,7] were included as an ordered response variable (‘no response’ = ‘0’, ‘turning-away response’ = ‘1’ and ‘erratic response’ = ‘2’; see Results for definitions), and moth ID was included as a random effect. The six pulse types, two moth sexes and seven sound levels were incorporated into the statistical models as fixed effects. The deviance between the full model and each model that lacked one of the explanatory variables outlined above was analysed. To test the effect of pulse type on the behavioural response, multiple comparisons were made for all combinations of pulse type and each *P*-value was then adjusted after controlling for the false discovery rate [39]. The effects of pulse type, sex and sound level on the overall pooled evasive actions and each individual avoidance response category were analysed with LR tests in generalized linear mixed models (GLMM) with a binomial error distribution using the ‘lme4’ package. The highest sound level that caused evasive responses in the greatest proportion of moths (100 dB SPL; see Results) was then compared with the other sound levels and the *P*-value was adjusted as above.

## Results

### Echolocation calls of *E*. *fuscus*

**Fig 1:**
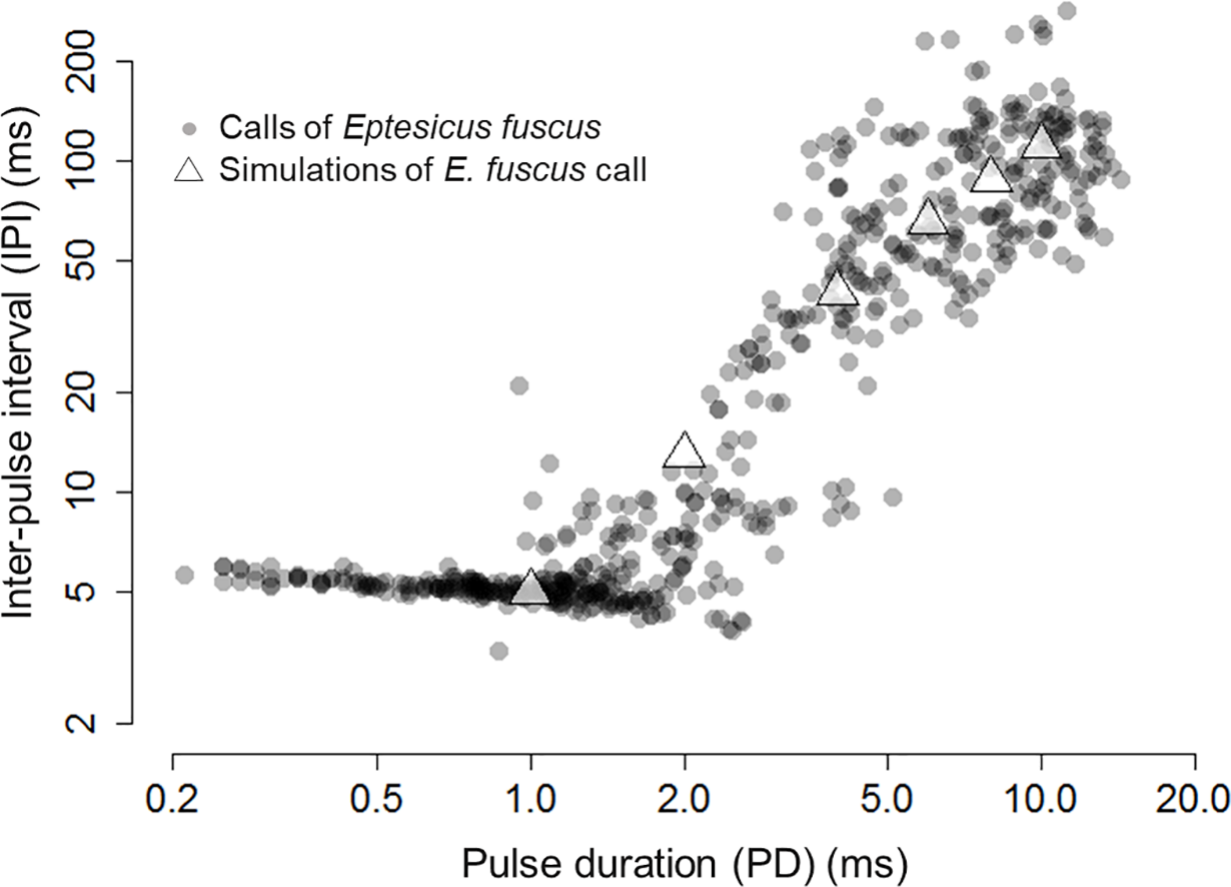
Temporal structures of echolocation pulses emitted by the big brown bat, *Eptesicus fuscus*. Circles denote the ultrasonic pulses of echolocating bats in a foraging sequence that were recorded in the field. Triangles represent the simulated ultrasonic pulses that were broadcast to moths during the behavioural experiment. Note that *E*. *fuscus* is currently a major insectivorous bat species in the area in which we collected the lucerne moth, *Nomophila nearctica*.

We successfully obtained 12 clear audio recordings of echolocation call sequences containing search to terminal phases from *E*. *fuscus* foraging aerially in space with low clutter, the temporal structures of which are shown in Fig 1. In these pulses, the PD ranged from 14.3 ms in the search phase to 0.2 ms in the terminal buzz (*N* = 599) and the IPI ranged from 286.2 ms in the search phase to 3.3 ms in the terminal buzz (*N* = 586). The search phase pulses had a mean duration of 9.0 ms (range = 3.5–14.3 ms; *N* = 111) and a mean IPI of 122.0 ms (range = 49.2–286.2 ms; *N* = 111); the approach phase pulses had a mean duration of 6.0 ms (range = 2.0–13.3 ms; *N* = 145) and a mean IPI of 54.3 ms (range = 9.8–118.2 ms; *N* = 145); and the terminal buzz had a mean duration of 1.3 ms (range = 0.2–4.3 ms; *N* = 339) and a mean IPI of 6.4 ms (range = 3.3–27.4 ms; *N* = 326). The six representative pulses that were simulated for the behavioural experiment (search, early-approach, middle-approach, late-approach, early-terminal and middle-terminal phases) are shown as white triangles in Fig 1 (see also S1 Data).

### Nocturnal activities of bats and moths

**Fig 2:**
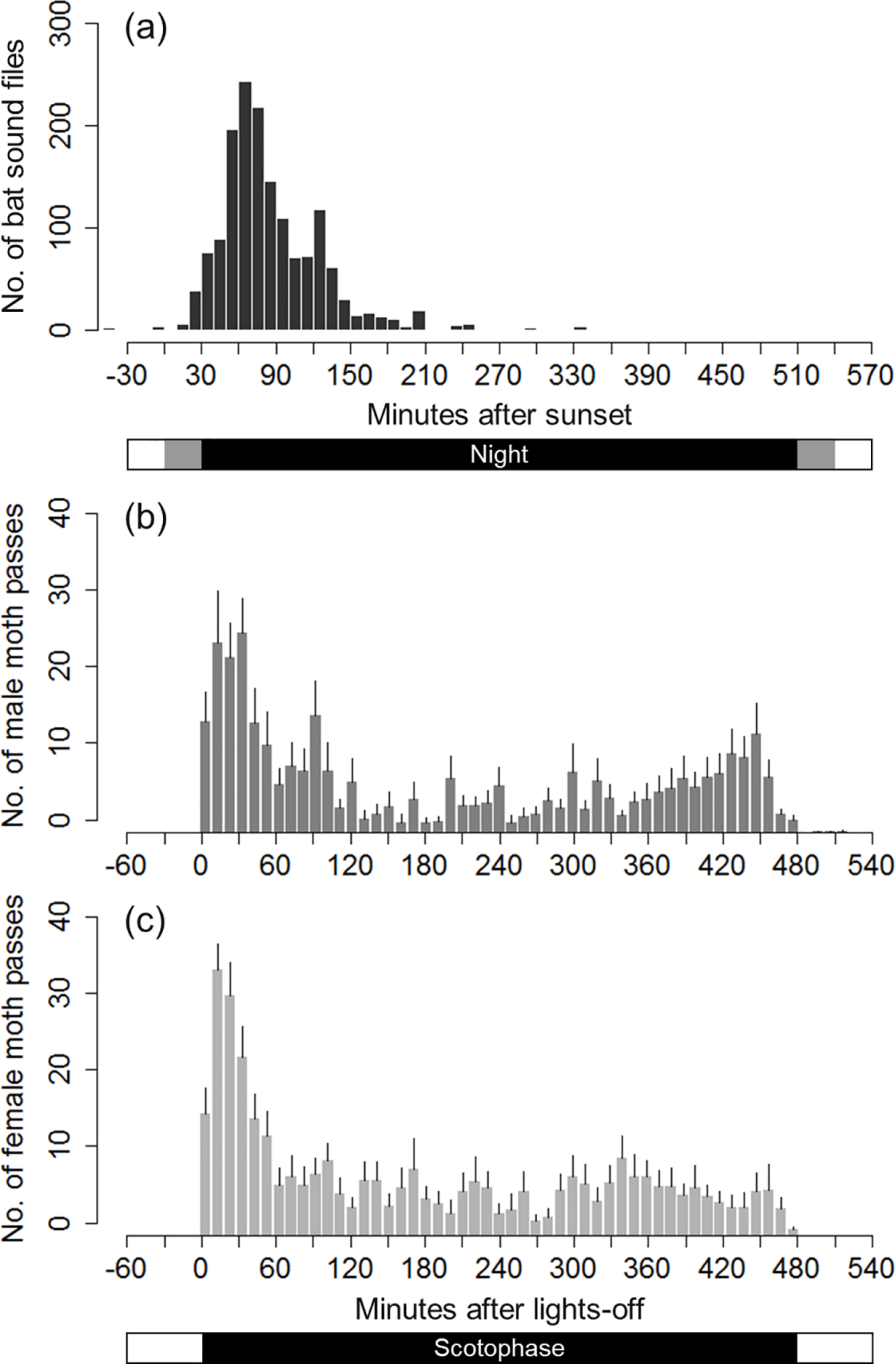
Active times of the bat *Eptesicus fuscus* and the moth *Nomophila nearctica*. (a) Early in the night, *E*. *fuscus* frequently echolocated in the field, with 1571 sound files being recorded over 14 clear nights in June to July 2014 and 2015. Bars beneath the *x*-axis denote daytime (white), twilight (grey) and nighttime (black) in early July in Ontario, Canada. (b, c) In the early scotophase (16L:8D), i.e. shortly after lights-off, both male (b) and female (c) *N*. *nearctica* exhibited active locomotion, with a standardised active rate (i.e. nocturnality; see Methods) of 98.6% for male (*N* = 16) and 99.2% for female (*N* = 18). Error bars in (b) and (c) indicate the standard error of the mean, and white and black bars beneath the *x*-axis denote photophase (daytime) and scotophase (nighttime), respectively, in the experimental room. Activity was significantly different between the photophase and scotophase.

To eliminate the possibility that *N*. *nearctica* is temporally isolated from *E*. *fuscus*, we examined the overlap in active times between the bat predator and moth prey. We obtained 681 audio files of echolocation calls of *E*. *fuscus* in the field in 2014 and 890 in 2015. We pooled the time stamps of these 1571 files and generated a histogram of the number of files in 10-min time bins (Fig 2A; S1 Data). Most of the files contained echolocation calls were recorded between 20 min after sunset (10 min before dusk) and 210 min after sunset (180 min after dusk, 330 min before sunrise or 300 min before dawn) at the study site in June and July, with peak activity (42.6% of the call files) occurring 50–80 min after sunset (20–50 min after dusk).

The recording of moth activity in the laboratory demonstrated high levels of nocturnality in both males (98.6%, *N* = 16) and females (99.2%, *N* = 18) (LR test in GLMM, photophase vs. scotophase, *χ*_*1*_^*2*^ = 19249, *P* < 0.0001) (Fig 2B and 2C; S1 Data). Furthermore, there was no significant difference in the time of activity between males and females (*χ*_*1*_^*2*^ = 1.55, *P* = 0.21), indicating that both sexes frequently moved in the early scotophase (first 2 h of scotophase). Assuming that the circadian rhythm of moths in the wild was maintained in the laboratory, Fig 2 indicates that both *E*. *fuscus* and *N*. *nearctica* are active early in the night, with the majority (93%) of bat echolocation calls being recorded 30–150 min after sunset, when 49% of moth activity occurred.

### Evasive manoeuvres

We tested that ultrasonic pulses of >60 dB SPL induced frequent evasive actions regardless of the attacking stage in the echolocation sequence. We categorised the behavioural reactions of flying *N*. *nearctica* to bat call simulations into three types: no response, turning-away response, which was defined as directional flights with courses away from the sound source, and erratic response, which was defined as flights with unpredictable trajectories, including looping-flight, zigzagging-flight and spiral-flight (S1 Video) [7,9,32,33,38]. Flight cessation (passive dive) was never observed in this species, which contrasts with noctuid, erebid, pyralid and geometrid moths [7,9,10,38,40–44].

**Fig 3:**
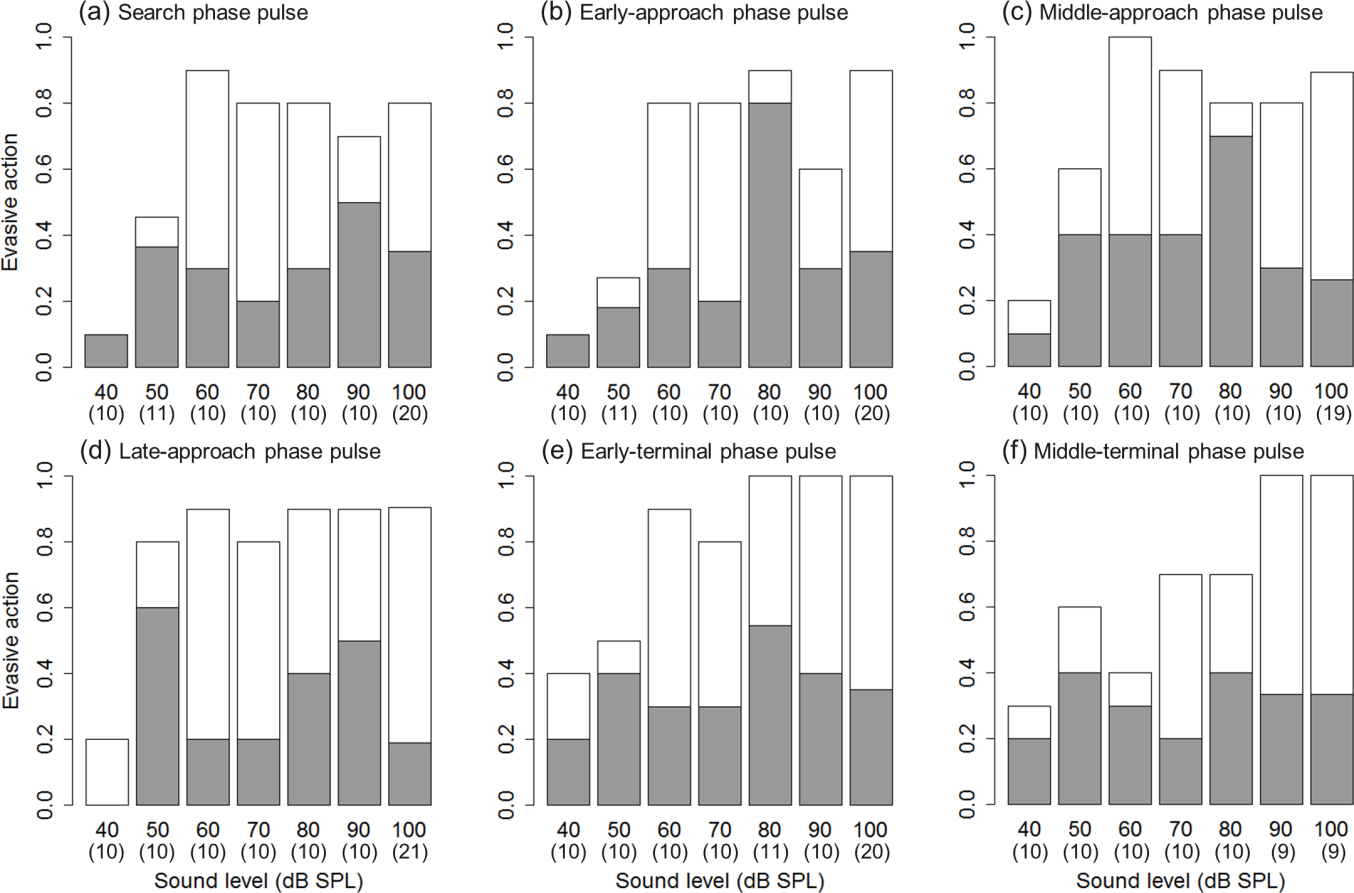
Mode of evasive action taken by flying *Nomophila nearctica* following the broadcast of a 30-kHz pure tone pulse with a similar temporal structure to the echolocation calls of *Eptesicus fuscus*. (a) Search phase pulse, (b) early-approach phase pulse, (c) middle-approach phase pulse, (d) late-approach phase pulse, (e) early-terminal phase pulse and (f) middle-terminal phase pulse. Grey bars indicate a turning-away response and white bars indicate an erratic response in the flying moth. Sample sizes are given in parentheses beneath the sound levels and the *y*-axis denotes the proportion of moths showing each type of response. Multiple comparisons among all combinations of pulse types showed that there was no significant difference in the evasive manoeuvres performed.

No significant differences were observed in the mode and incidence of evasive actions among the six pulse types that were broadcast at 40–100 dB SPL (LR test in CLMM; *LR statistic* < 8.00, adjusted *P* > 0.070) (Fig 3; S1 Data). There were also no differences between the sexes in the evasive manoeuvres performed (*LR statistic* = 0.15, *P* = 0.70). However, sound levels did significantly affect the evasive manoeuvres (*LR statistic* = 54.90, *P* < 0.0001).

**Fig 4:**
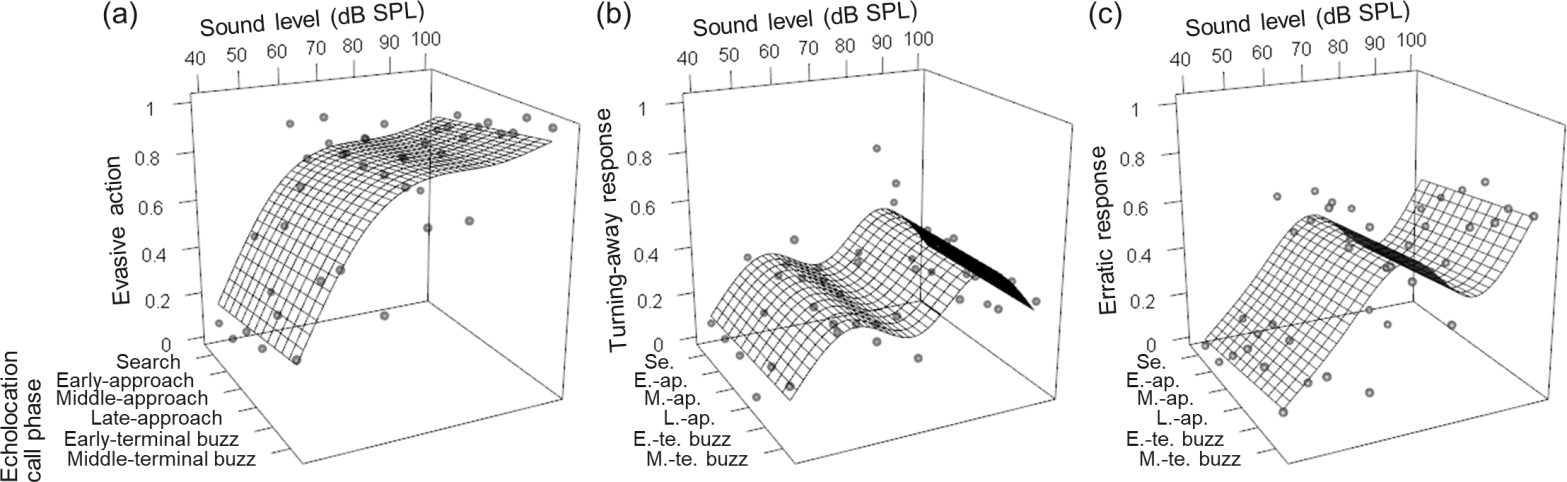
Effect of sound level on the evasive responses of flying *Nomophila nearctica*. (a) The proportion of pooled turning and erratic responses significantly differed among sound levels. (b) The proportion of turning responses did not differ among sound levels. (c) The proportion of erratic responses significantly differed among sound levels. The same dataset was used as in Fig 3. Circles identify the averages and the lattice represents the average spline deduced by generalized additive models. ‘Se.’ to ‘M.-te. Buzz’ in (b) and (c) are abbreviations of the echolocation call phases shown in (a).

Stimuli that were presented at 100 dB SPL elicited the highest rate of pooled escape responses (91%; cf.22% for 40 dB SPL,53% for 50 dB SPL,82% for 60 dB SPL,80% for 70 dB SPL,85% for 80 dB SPL,83% for 90 dB SPL) (Fig 4; S1 Data). There was no significant difference in the rate of overall evasive actions, i.e. the sum of the turning-away responses and erratic responses, between 100 dB SPL and 60, 70, 80 and 90 dB SPL (LR test in GLMM; *χ*_*1*_^*2*^ < 4.45, adjusted *P* > 0.070) (Fig 4A). However, stimuli that were presented at 40 and 50 dB SPL induced significantly fewer escape responses than those presented at 100 dB SPL (*χ*_*1*_^*2*^ > 32.16, adjusted *P* < 0.0001) (Fig 4A).

We did not find any significant difference in turning-away responses alone with increasing sound level (LR test in GLMM; *χ*_*1*_^*2*^ < 6.49, adjusted *P* > 0.065) (Fig 4B) and the erratic responses alone also did not significantly differ between 100 dB SPL and 60, 70 and 90 dB SPL stimuli (LR test in GLMM; *χ*_*1*_^*2*^ < 3.02, adjusted *P* > 0.12). However, the 40, 50 and 80 dB SPL stimuli induced significantly lower erratic responses than the 100 dB SPL stimuli (*χ*_*1*_^*2*^ > 10.08, adjusted *P* < 0.0030) (Fig 4C).

## Discussion

In the present study, we found that flying *N*. *nearctica* exhibited both turning-away and erratic flight in response to fairly quiet (60 dB SPL) bat-like ultrasound stimuli, regardless of the temporal structures of the echolocation pulses. This sound level corresponds to echolocation calls emitted by *E*. *fuscus* at more than 20 m away [28,45,46], from which distance the observed evasive manoeuvres could allow the moths to escape to a safety zone before the bat approaches, making them crucial for avoiding bat predation. The auditory cells of *N*. *nearctica* can respond to search phase pulses of *E*. *fuscus* at >48 dB SPL, approach phase pulses at >51 dB SPL and the terminal buzz at >53 dB SPL [28], implying that any observed evasive actions in response to stimuli of up to 40 dB SPL were simply response-like behaviours. Although we focused on the defensive behaviour of a single moth prey (*N*. *nearctica*) against a single bat predator (*E*. *fuscus*) in one location, both neural [28] and behavioural data (this study) support our observation that moths exhibit early escape strategies in response to >60 dB SPL ultrasound stimuli. The response-inducing sound levels and pulse structures indicate that *N*. *nearctica* escapes bat predators both while other flying insects are being chased (and subsequently captured) by the bats and while the bats are approaching and representing a real predation threat [7,33]. The emission of warning or jamming signals by other moths species, such as tiger moths, helps them to avoid being captured after their accurate localisation by a bat [10,14]. However, sound generation has not been found in *N*. *nearctica*.

Diving and dropping to the ground is likely to be the ultimate last-ditch response of moths to a predation threat, being performed shortly before they would be captured by the bat [33]. However, we did not observe passive diving in *N*. *nearctica* and flight cessation has never been observed in this moth or hedylid moths (nocturnal butterflies) [47]. Similarly, tethered Asian corn borer moths, *Ostrinia furnacalis* (which are in the same family as *N*. *nearctica*), do not exhibit a substantial number of passive dives but evasive action is evoked by 62-dB ultrasonic pulses in ca. 80% of the individuals [48]. Therefore, since *O*. *furnacalis* and *N*. *nearctica* have similar auditory thresholds and frequency-tuning curves [28,49], it seems likely that early avoidance behaviours are a common counter-measure in crambid moths against predacious bats. By contrast, noctuid (two auditory cells in individual ear) [7,9], erebid (two cells) [38], notodontid (one cell) [50], geometrid (four cells) [7] and pyralid (four cells) [42,44] moths show flight cessation behaviour upon the detection of approach-phase echolocation pulses of bats. Therefore, it appears that flight cessation does not depend on the number of auditory cells, since crambid moths have four cells in each ear, like pyralid and geometrid moths [4,6,11].

Intense ultrasound evokes flight cessation and erratic behavioural responses in noctuid moths [9]. However, in *N*. *nearctica*, relatively quiet ultrasonic pulses (60 dB SPL) induced turning-away and erratic flight, whereas even intense ultrasound stimuli did not evoke flight cessation (Figs 3 and 4). It is risky to cease flight in proximity to an approaching bat because bats can track and often catch diving moths [10]. The behavioural data obtained in this study suggest that one of the optimal evasion tactic for flying *N*. *nearctica* is to fly away early to avoid being locked onto and chased by a bat. Although Krauel and the colleagues [31] state that *N*. *nearctica* can fly high for the autumn migration, we frequently observed this moth flying close to the ground or grasses in summer, suggesting that the observed erratic response allows low-flying moths to escape from bats [20,21,51].

*Nomophila nearctica* is abundant in North America, where it is commonly seen and easily collected in summer [29], and the active time of *N*. *nearctica* overlaps with that of *E*. *fuscus* (Fig 2). However, DNA fragments of *N*. *nearctica* have not been detected from the faeces of *E*. *fuscus* and *M*. *lucifugus* [52,53], suggesting that wild bats rarely consume *N*. *nearctica* in Canada. Thus, since noctuid moth species that are captured by bats do not show erratic flight in response to relatively quiet ultrasound stimuli, it appears that *N*. *nearctica* can successfully escape insectivorous bats through a combination of early turning away from cruising bats and erratic flights against hunting bats at a distance.

## Supporting information

S1 Data All raw data for Figs 1–4. (XLSX)

S1 Video Examples of the three behavioural responses (no response, turning away and erratic flight) of *Nomophila nearctica* to an early-approach phase pulse broadcast at 90 dB sound pressure level (SPL). (MP4)
